# Progress and Challenges of Understanding Cardiorenal Syndrome Type 3

**DOI:** 10.31083/j.rcm2401008

**Published:** 2023-01-04

**Authors:** Raquel Silva Neres-Santos, Giovana Marchini Armentano, Jéssica Verônica da Silva, Carlos Alexandre Falconi, Marcela Sorelli Carneiro-Ramos

**Affiliations:** ^1^Laboratory of Cardiovascular Immunology, Center of Natural and Human Sciences (CCNH), Federal University of ABC, 09210-170 Santo André, SP, Brazil

**Keywords:** cardiorenal syndrome type 3, acute renocardiac syndrome, heart failure, acute kidney injury, acute cardiac injury, epigenetics

## Abstract

The pathologies of the kidney and heart have instigated a large number of researchers around the world to try to better understand what the exact connectors responsible for the emergence and establishment of these diseases are. The classification of these pathologies into different types of cardiorenal syndromes (CRSs) over the last 15 years has greatly contributed to understanding pathophysiological and diagnostic aspects, as well as treatment strategies. However, with the advent of new technologies classified as “Omics”, a new range of knowledge and new possibilities have opened up in order to effectively understand the intermediaries between the kidney-heart axis. The universe of micro-RNAs (miRNAs), epigenetic factors, and components present in extracellular vesicles (EVs) have been protagonists in studying different types of CRSs. Thus, the new challenge that is imposed is to select and link the large amount of information generated from the use of large-scale analysis techniques. The present review seeks to present some of the future perspectives related to understanding CRSs, with an emphasis on CRS type 3.

## 1. What is Known about CRS 3? 

The definition of cardiorenal syndrome (CRS) highlights the bidirectional nature of heart-kidney interactions and is classified into 5 clinical subtypes based on the organ (heart or kidney) and the progression time course (acute or chronic) [1]. Impairment of cardiac and/or renal functions can cause injury or dysfunction of these organs later [2] resulting in a cascade of feedback mechanisms causing damage to both organs.

CRS type 1 (CRS 1) typically occurs because of an acute heart condition such as heart failure (HF), often following an ischemic or non-ischemic disease. CRS type 3 (CRS 3) also occurs acutely, but originates from renal dysfunction characterized by an acute kidney disease leading to acute HF. Since heart and chronic kidney disease often occur simultaneously, CRS types 2 (CRS 2) and 4 (CRS 4) are often related [3, 4]. CRS 2 differs in that it starts with chronic HF leading to kidney failure, whereas CRS 4 originates from chronic kidney disease causing subsequent HF. CRS type 5 (CRS 5) includes concomitant renal and cardiovascular disease caused by systemic disease (such as obesity, diabetes, metabolic syndrome, hypertension) since it presents the simultaneous involvement of the kidneys and the heart through organ damage or dysfunction (Table 1, Ref. [2, 4, 5, 6, 7, 8, 9, 10, 11, 12, 13, 14, 15]).

**Table 1.** **Classification of the 5 subtypes of CRS**.

Classification	Category	Start	Target	Description	References
Type 1	Acute CRS	Heart	Kidney	Impaired cardiac output reduces glomerular filtration rate by increasing venous pressure leading to acute kidney injury.	[4, 5, 11]
Type 2	Chronic CRS	Heart	Kidney	Hypoxia and low cardiac output in chronic heart failure increase sympathetic nervous system activity, activate the RAAS, increase renal oxidative stress leading to renal fibrosis, functional loss, and permanent chronic kidney damage.	[6, 7, 15]
Type 3	Acute RCS	Kidney	Heart	Renal failure generates excessive hemodynamic pressure, responsible for left ventricular hypertrophy (LVH) that triggers the syndrome’s heart problems. Leukocyte accumulation and increase in proinflammatory cytokines in acute renal failure lead to often fatal cardiac myocyte apoptosis.	[8, 9, 14]
Type 4	Chronic RCS	Kidney	Heart	Chronic kidney disease itself leads to cardiovascular disease with coronary atherosclerosis or ventricular hypertrophy usually caused by the effect of toxins, metabolic, cellular and hormonal factors.	[10, 11, 12]
Type 5	Secondary CRS	Both	Both	Diseases such as hypertension, diabetes mellitus and sepsis cause damage to pathophysiological changes in cardiac and renal function.	[2, 13]

The table summarizes all known SCR types with general considerations. Each subtype has different etiologies (start and target) and categories (acute or chronic). CRS, Cardiorenal syndrome; RCS, Renocardiac syndrome; RAAS, Renin-angiotensin-aldosterone system.

Regarding the clinical aspects of CRS 3, it is established that acute kidney injury (AKI) is capable of leading the heart to serious and acute injury, resulting in arrhythmias, acute compensated HF, acute coronary syndrome, cardiac hypertrophy as results of electrolyte imbalance, potassium and calcium abnormalities levels, fluid overload, metabolic acidosis, intensity immune response and atherosclerosis [16]. The cardiac dysfunction response may be noticed in all stages of AKI, and are prominent in stage 3 [17]. Besides the strict relationship between AKI and cardiac dysfunction in the intensive care units AKI patients, early diagnosis in order to avoid further cardiac complications still a challenge [18].

CRS develops through hemodynamic and non-hemodynamic mechanisms. The hemodynamic abnormality was the first mechanism reported in the CRS. It is clear that HF, with or without preserved ejection fraction, leads to renal hypoperfusion, elevating kidney venous pressure, and worsening renal function [19]. The non-hemodynamic mechanisms include the sympathetic nervous system (SNS), renin-angiotensin-aldosterone system (RAAS), oxidative stress, and inflammation. Kidney and HF in CRS activate the RAAS and SNS, which are the main cardiorenal connectors [4]. The hyperactivation of SNS is a compensatory mechanism in CRS that the exacerbated the release of catecholamines. Moreover, the excessive release of neurohormones also decreases the β1:β2 ratio and impairs heart function [20, 21]. The RAAS exerts an important role in CRS by reducing renal perfusion in response to increased levels of angiotensin II (Ang II) and aldosterone. Ang II can directly affect renal hemodynamics by inducing the synthesis of proinflammatory cytokines, regulating cell proliferation, pro-fibrotic factors, and cell death. In the cardiovascular system, Ang II may induce vascular hypertrophy and endothelial dysfunction, whereas aldosterone promotes cardiac hypertrophy, cardiac dilatation, and HF through mineralocorticoid receptors localized in cardiomyocytes and fibroblasts [22, 23, 24]. In addition, data from the literature suggest that fibrosis may be one of the main pathophysiological factors of CRS as it acts as a common biomarker of inflammation and oxidative stress [6, 7]. Mitochondria dysfunction is a key element in both cardiac damage and kidney injury by the massive mitochondria disruption resulting in increased apoptosis of proximal tubules epithelial tubular cells, and in a short time of kidney injury, and cardiac tissue showed the same intensive mitochondria fragmentation and death of cardiomyocytes [16, 25]. Oxidative/nitrosative stress also was reported as a contributor to both renal and cardiac damage through mitochondrial and extra-mitochondria sources, especially nicotinamide adenine dinucleotide phosphate oxidases and depletion of antioxidant endogenous defense [26, 27].

Regarding experimental approaches, some rodent models (rats and mice) are widely used to study CRS through induced cardiac or renal dysfunction. The possibility of introducing targeted genetic mutations makes animal studies possible and feasible. In addition, some features are common in both HF and chronic kidney failure, such as oxidative stress, inflammation or fibrosis leading to organ remodeling and dysfunction. As an example of CRS 1, several research groups have injected potassium chloride into mice simulating acute cardiac dysfunction, generating cardiac arrest and subsequent cardiopulmonary resuscitation. This leads to AKI, with a decrease in the estimated glomerular filtration rate (eGFR) together with an increase in serum creatinine levels [7]. As a CRS 2 model, chronic cardiac injury through sudden occlusion of the descending coronary artery causes myocardial infarction (MI). This leads to left ventricular dilatation, renal fibrosis, reduced eGFR, and elevated serum creatinine [8]. In relation to CRS 3, the most used model to induce is the renal ischemia-reperfusion model in which pinching the renal pedicle causes damage by hypoxia, compromising circulation and oxygenation and causing renal dysfunction. Then, it is possible to observe cardiac alterations, such as cardiac hypertrophy, cardiac electrical disturbances, and apoptosis [28]. AKI in CRS 3 activates the SNS and the RAAS, in addition to systemically activating the immune system [29]. Increased activation of immune cells, such as activation of lymphocytes, monocytes, and endothelial cells, is caused as cells killed by AKI activate damage-associated molecular patterns (DAMPs) via the inflammasome and toll-like receptors (TLRs). Such activated receptors release pro-necrotic and pro-inflammatory cytokines generating regulated cell death. In addition, caspases involved in the apoptosis pathway have increased activity generating cardiac hypertrophy after renal ischemia reperfusion injury, indicating stimulation of apoptosis independently from IL-1β [30].

Although many studies on CRS use animal models to capture the interaction between organs, it is impossible to extrapolate the results to the human condition, mainly due to interspecies differences. Thus, there is a need for better models such as *in vitro* options which mimic dynamic organ-organ crosstalk to understand how hemodynamic, biochemical, and hormonal factors contribute to develop CRS. Furthermore, it is necessary to not only study the cell-cell interaction, but also the physical environment generated by the factors which are secreted by the extracellular matrix and by the cells themselves. Therefore, 3D co-culture has been gaining ground in pathophysiological studies of cardiomyocytes, but it still needs to be more used [10].

In addition to the aforementioned mechanisms, the accumulation of uremic toxins in the body (uremia) in kidney diseases is expected, and the cardiac implications have been reported in a large number of papers. Uremic conditions enable retaining solutes, including small water-soluble compounds (molecular weight <500 Da), medium molecules (molecular weight >500 Da), and protein-bound solute toxins (i.e., Indoxyl sulfate (IS) and P-cresyl sulfate (PCS)) [14, 31]. Protein-bound uremic toxins are perhaps the toxins with the greatest deleterious effect on organ systems. Their low molecular weight and binding to serum albumin do not allow the elimination of these toxins by either normal or dialysis routes. Within this group of toxins, IS and PCS appear as two of the main toxins linked to CRS [15, 32].

IS and PCS are related to redox unbalance by increasing reactive oxygen species (ROS) in the kidney and heart, as well as cardiorenal fibrosis. In addition, these toxins contribute to increase kidney injury biomarkers such as renal kidney injury molecule-1 (KIM-1). IS promotes a proliferation of vascular smooth muscle cells, and there are also reports that IS may contribute to vascular stiffness, calcification, and ossification, which are common chronic kidney disease (CKD)-associated vascular abnormalities. Thus, both IS and PCS circulating levels correlate with vascular/aortic calcification in various stages of CKD [31]. Both are linked to renal failure progression and an increase of cardiovascular events, in addition to being associated with all-cause mortality [33]. Thus, these compounds can cause renal inflammation and fibrosis, increased cardiac protein and collagen synthesis, as well as endothelial dysfunction [34].

Over all phases of AKI, uremic toxins may be concentrated in consequence of the declining in eGFR, decrease on anion transporters 1 and 3 (OAT 1/3), alterations in metabolic prolife, and dysbiosis of gut microbiote, favotering the accumulation, synthesis and release uremic toxins [35, 36]. An unilateral ischemia and reperfusion injury (UIRI) model in mice demonstrated the accumulation of IS in the early stage, associated with reduction of OAT1 [37]. Uremia by IS and PCS was assessed recently in septic AKI patient, according RIFLE classification, in which IS, PCS and serum creatinine was elevated in first day of hospitalization. However IS and PCS serum levels decreased along the subsequent days, specifically in the risk and injury AKI stages. In comparison with CKD patients, the levels of these two uremic toxins were lower in AKI patients [16, 36]. Even though such accumulation in the AKI has been indicated in some reports, there is a necessity to assess whether uremic toxins would be capable of providing detrimental effects on the heart in the early phases of kidney disease, moreover in CRS 3.

**Fig. 1.**
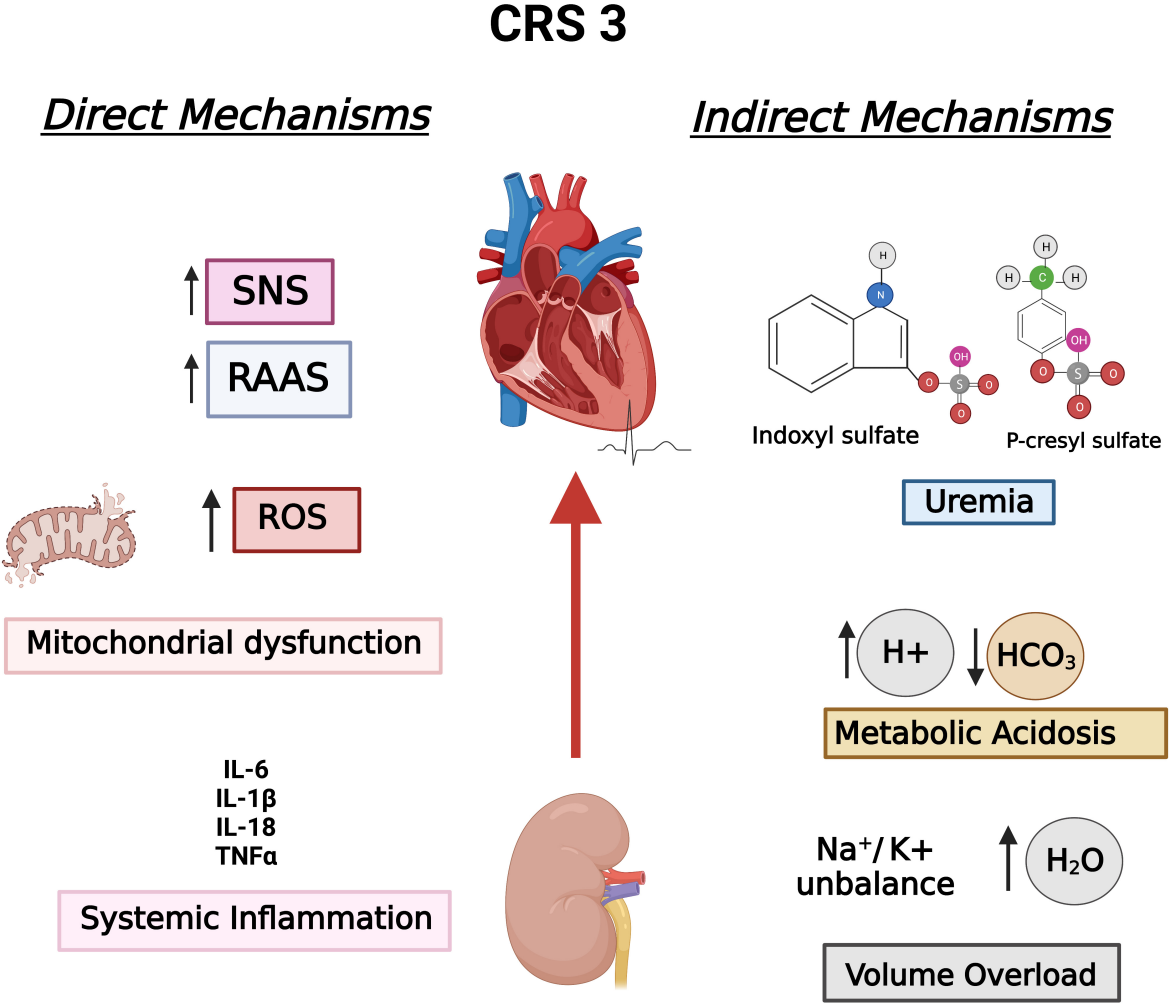
**CRS 3 established pathophysiological mechanisms divided into direct mechanisms and indirect mechanisms, according to Di Lullo *et al***. Hydroelectric unbalance, metabolic acidosis, and uremia are consequences of impair or loss of renal function, which are considered indirect mechanisms, whereas SNS, RAAS, mitochondrial dysfunction, and inflammation affect both organs, comprising direct mechanisms. CRS 3, Cardiorenal syndrome type 3; RAAS, Renin-angiotensin-aldosterone system; ROS, Reactive oxygen species; SNS, Sympathetic nervous system.

Fig. 1 summarizes the aforementioned mechanisms in the context of CRS 3, classifying them into indirect and direct mechanisms according to Di Lullo *et al*. [16, 15].

## 2. Frontiers in CRS3 Understanding: The Non-Coding RNAs Universe and Epigenetics Factors

An exponential number of papers have reported direct and indirect mechanisms responsible for cardiac outcomes after AKI since the five CRS classifications creation in 2008; however, the complete mechanisms related to CRS 3 development and a deep understanding of the pathological course are still unknown, demonstrating the necessity to find out new key biomarkers and pathways [15, 38]. In this sense, Virzìet al. [39] published a review focused on the recent alternatives which may contribute to the progression of CRS, epigenetics and non-coding RNAs (ncRNAs), and extracellular vesicles (EVs), as well as new technological approaches to a better comprehension of such contribution, the omics field (proteomic, metabolomic, transcriptomic). Since these emerging molecules and technology are promising, it is crucial to revise the recent discoveries involving epigenetics, ncRNAs, and omics analysis in the AKI, cardiac hypertrophy and dysfunction, and finally CRS 3. ncRNAs have received a simple classification considering the molecule size: small ncRNAs and long ncRNAs. microRNAs (miRNAs) are part of small ncRNAs, containing about 22 nucleotides. miRNAs are involved in the regulation of transcription by off-targeting messenger RNAs (mRNAs). Long non-coding RNAs (lncRNAs) and circular RNAs (circRNAs) compose long ncRNA classification, having more than 200 nucleotides and exerting a variety of functions that are distinct according to cellular position (nucleus or cytoplasm), such as regulating the expression of mRNAs and miRNAs, acting like miRNAs sponges, and remodeling of chromatin. Similar lncRNAs, circRNAs sponge miRNAs, enhancing gene expression, act as a scaffold for transcription factors both in the cytoplasm or shuttling target genes, and possibly encode proteins [40, 41].

Epigenetics comprises a regulatory mechanism of gene expression without affecting the DNA nucleotide sequence. The most common types of epigenetic mechanisms are modification of histones by acetylation, methylation and phosphorylation, and DNA methylation. Histones may receive a negatively charged acetyl group (acetylation) by histone acetyltransferases (HATs) enhancing gene expression or lose acetyl groups by histones deacetylases (deacetylation) suppressing gene expression [42]. In histone methylation, one or multiple methyl groups may be inserted by histone methyltransferases (HMTs), whereas histone demethylases (HDMs) enzymes are responsible for removing these groups [43]. DNA methylation leads to the suppression of gene expression by enzyme DNA methyltransferases (DNMT) by adding a methyl group to 5’ carbon of cytosine presented in the cytosine-phosphor-guanine (CpG) dinucleotide sites forming 5-methylcytosine (5mC). In turn, DNA demethylation may occur when DNMT1 is depleted or absent, by DNA methylation erasers, or by ten-eleven translocation (TET), actively converting 5mC to 5-hydroxymethylcytosine (5hmC) [44, 45].

### 2.1 ncRNAs in the AKI, Cardiac Hypertrophy, and CRS

ncRNAs have been reported to participate in pathophysiology and repair in the case of AKI, and are additionally promising biomarkers and therapeutic targets [46]. miRNAs are the greatest studied ncRNAs in the AKI by distinct pathways. It was identified that mitochondrial quality control processes in the AKI may be controlled by miRNAs. Recent research revealed that miR-140-5p expression was repressed by hypoxia inducible factor 1a (HIF-1α) in ischemic AKI, leading to an increase in PARKIN and consequently mitophagy [47]. miR-214 is capable of targeting mitofusin-2 (Mfn2), and leading to massive mitochondrial fragmentation, ATP synthesis depletion, and apoptosis in renal proximal tubular cells in the ischemic kidney [48]. Exosomal miR-19b-3p and miR-155 are exchanged between M1 macrophages and tubular cells, resulting in the impairment of ischemic kidney injury by suppressing cytokine signaling‑1 (SOCS1), a negative regulatory factor of nuclear factor kappa beta (NF‑κB) [49, 50]. miR-182 is studied in AKI as a consequence of promoting apoptosis by different pathways [51, 52]. Differentially, some miRNAs are characterized by protecting against AKI. miR-21 is often reported as a renoprotective miRNA and highly expressed during AKI, targeting key mechanisms of inflammation, autophagy, apoptosis, and fibrosis [53, 54, 55]. HIF-1α was indicated as an upstream effector of miR-21 [40]. More recently, the same overexpression of miR-21 was shown by Pushpakumar *et al*. [56], but in aged-AKI associated with renal ischemic and reperfusion models. Like miR-21, other miRNAs were suggested to be protectors against AKI. miR-688 has 124 gene targets, among them mitochondria protein 18 (MTP18), which is involved in mitochondrial fragmentation. In the AKI MTP18 is suppressed, leading to mitochondria dynamic preservation, and avoiding tubular cell apoptosis [57]. miR-205 is down-regulated in AKI, but the administration of miR-205 mimic provides an anti-apoptotic effect, but differently from miR-688, its effect is mediated by PTEN/Akt pathway [58].

On the other hand, lncRNAs are lesser studied than miRNAs; however, there is an increasing quantity of studies shedding light on the contribution of these ncRNAs in AKI. Metastasis-associated-related lung adenocarcinoma transcript 1 (MALAT1) is highly conserved and one of the first lncRNAs linked to diseases, and was detected overexpressed in the plasma and kidney samples in humans, animals, and cells. However, knockdown of MALAT1 only inhibited apoptosis of tubular epithelial in septic AKI, promoting the overexpression of miR-146a, a negative regulator of NF-κB [59, 60]. Geng *et al*. [61] reported that lncRNA Growth Arrest Specific 5 (GAS5) exerts an apoptotic effect in ischemic AKI, acting as a competing endogenous RNA for miR-21 and leading to overexpression of the antiangiogenic, pro-inflammatory, and pro-apoptotic thrombospondin 1 (TSP1). Currently, both *in vivo* and *in vitro* models indicated that the lncRNA maternally expressed 3 (MEG3) was upregulated during ischemic AKI, targeting miR-129-5p, and elevating the expression of high-mobility group box-1 (HMGB1), a protein intensively released by necrotic tubular epithelial cells in the AKI, conducting inflammation and apoptosis as well [62].

In relation to cardiac complications, a wide range of ncRNAs also play in the development of cardiac hypertrophy and HF as demonstrated by a large number of *in vivo* or *in vitro* hypertrophy models. miR-17-5p, miR-29a, miR-100-5p, miR-128, miR-199a, and miR-302-367 clusters were reported to regulate pathological autophagy during cardiac hypertrophy, abrogating the expression of Mfn2, regulating and stimulating the PTEN/Akt/mTOR pathway, directly targeting mTOR, and activating the hypertrophic GSK3β/mTOR pathway [63, 64, 65, 66, 67, 68]. In addition to autophagy, miRNAs regulate other cellular processes involved in cardiac remodeling, such as metabolism and mitochondrial integrity. miR-24, miR 27b-3p and miR-214 were discovered to be overexpressed and promote mitochondrial dysfunction and oxidative stress during distinct cardiac hypertrophy models by targeting glycose-6-phosphate dehydrogenase (G6PD), PCG1α/PCG1β and sirtuin 3 (SIRT 3) [69, 70, 71]. miR-223 also contributes to cardiac hypertrophy by targeting the apoptosis repressor with caspase recruitment domain (ARC), which inhibits the activation of mitochondrial permeability transition (MPT) [72].

Other miRNAs may be called pro-hypertrophic miRNAs because of regulating key cardiac components during hypertrophy. miR-339-5p contributes to cardiomyocyte hypertrophy by depleting valosin-containing protein (VCP), in turn promoting activation of the mTOR/S6K pathway, an important sarcomeric protein synthesis pathway [73, 74]. miR-208a and miR-208b are only expressed in the heart together with MCH and β-myosin heavy chain (β-MHC). It was demonstrated that miR-208 contributes to cardiac remodeling by suppressing two negative regulators of hypertrophy, thyroid hormone–associated protein 1 (Thrap1) and myostatin [75]. The overexpression of miR-208a and its pro-hypertrophic properties was reported in diabetic cardiomyopathy [76]. miR-195 was revealed to be up-regulated during cardiac hypertrophy [77], and a study proposed that high mobility group A1 (HMGA1) was the target with a prominent elevation of atrial natriuretic factor (ANF) expression [78]. Moreover, miR-195 also may induce cardiac arrhythmia during hypertrophy [79]. It was proposed that miR-195-5p mediated hypertrophic response in H9c2 cells treated with angiotensin II (Ang II), depleting Mfn2 and F-box and WD-40 domain protein 7 (FBXW7). FBXW7 has anti-hypertrophic properties [77, 80]. Different studies have indicated the overexpression of miR-21 in cardiac hypertrophy and HF [81], however it has shown dual mechanisms, being either detrimental or beneficial according to cell type [82].

Over the few years, CRS studies demonstrated that ncRNAs are promising mechanisms to be deeply studied. miR-21 has been demonstrated to be a potential target. Wang *et al*. [83] conducted a clinical study to assess whether miR-21 could be a diagnostic and prognostic biomarker in CRS 2 patients compared with traditional biomarkers (e.g., C-cystatin, KIM-1, and N-terminal proBNP (NT-proBNP)). The results showed that miR-21 and the well-known kidney and heart biomarkers were increased in plasma samples, but miR-21 had a medium diagnosis value per se, while miR-21 and C-cystatin together had a significant diagnostic value [83]. Di *et al*. [84] reported that renal tubular epithelial cells stimulated with transforming growth factor beta (TGFβ) released a large number of EVs containing miR-21, and cardiomyocytes treated with these vesicles presented a hypertrophic response. miR-21 was also upregulated in a cardiac hypertrophy model induced by uremia, in which both miR-21 and miR-29 were downstream of angiotensin-converting enzyme (ACE) and angiotensin receptor 1a (ATR1) [85]. Despite all of these findings, studies clarifying whether ncRNAs may be involved in CRS 3 do not yet exist. Considering the wide range of ncRNAs promoting renal and cardiac diseases and the probability of finding them in circulation, it is possible to presume that these nucleic acids likely participate in kidney-heart crosstalk.

### 2.2 Epigenetic Aberrations in the AKI, Cardiac Remodeling and CRS 3

Histone acetylation is the most evaluated epigenetic mechanism in AKI. Both histone acetylation and deacetylation may negatively or positively contribute to AKI pathophysiology according to the stimuli. In a model of 30 minutes of unilateral renal ischemia and reperfusion (IR) injury, Zage *et al*. [86] reported a time-dependent increase in H3 acetylation associated with overexpression of pro-inflammatory cytokines and pro-fibrotic genes. Additionally, a gender-specific IR injury model developed by Kim *et al*. [87] showed increased H3 acetylation mediated by the reduced negative regulation of plasminogen activation inhibitor 1 (PAI-1) conferred by HDAC11. An opposite outcome was observed by Ruiz-Andres *et al*. [88] when the H3 deacetylation of PCG-1α was mediated by the activation of NF-κB through TWEAK (a member of the TNF superfamily), leading to the attraction of HDAC. Moreover, PCG-1α depletion worsened AKI. The same deacetylation was reported by Li *et al*. [89] through the attraction of HDAC1 to the IL6 and IL12b promoter region mediated by activating transcription factor 3 (ATF3) during IR injury. Class III deacetylase SIRT3 was associated with enhanced AKI by reducing superoxide dismutase 2 (SOD2) and p53, and autophagy [90, 91].

Although DNA methylation data in AKI is still limited, it has been demonstrated that this epigenetic mechanism is altered or aberrant in kidney injury. In an *ex vivo* experiment of cold kidney ischemia for 24 hours without reperfusion and followed by 2 hours of reperfusion, Pratt *et al*. [92] reported for the first time that CpG sites of IFN-γ response element in the *C3 gene promotor* were remarkably demethylated in both groups compared to the control group. Two years later, the same research group reported that demethylation remained 6 months after the renal IR in the C3 gene promoter corresponding to NF-κB binding sites and IL-1/IL-6 response element sites [93]. Another study conducted by Zhao *et al*. [94] reinforced that the global, gene promotor, exons, and introns 5hmC level was significantly reduced in ischemic kidney disease, along the 1 and 7 days of reperfusion. Two other studies with AKI and transplanted patients pointed out that methylation status may be a biomarker in AKI patients through the presence of hypermethylation of kallikrein1 (KLK1) and calcitonin [95, 96].

Conversely demethylation, another study showed the presence of hypermethylation in genes responsible for avoiding AKI worsening and progression to CKD. Chou *et al*. [97] induced AKI in C57Bl/6 mice by right kidney nephrectomy and then ischemia in the left kidney after 2 weeks. The results demonstrated activation of pericytes, or myofibroblast, from 1 day to 14 days of renal reperfusion, which led to elevated expression of α-smooth muscle actin (α-SMA), a specific myofibroblast marker, by the hypermethylation of α-actin 2 repressor Ybx2. Myofibroblasts are key contributors to kidney fibrosis after AKI [97]. A different fibrotic kidney injury model induced by unilateral ureteral occlusion in mice was carried out by Yin *et al*. [98]. They observed that the fibrotic kidneys presented hypermethylation of Klotho due to overexpression of DNTM1 and DNTM3 promoted by TGF-β [98]. All these reports indicated that altered DNA epigenetics is crucial to the development of AKI, inducing the expression of genes that contribute to the aggrievement of injury and lead to chronic kidney disease.

Histone acetylation was first highlighted regarding cardiac hypertrophy and HF, and HAT and HDAC activities were indicated to favor or inhibit cardiac hypertrophy, depending on the targets. Zhang *et al*. [99] demonstrated that the cardiac-specific class II HDACs, HDAC9 or MIRT, and HDAC 5 had anti-hypertrophy effects by inhibiting the expression of myocyte enhancer factor-2 (MEF2), a transcription factor of pro-hypertrophy genes like ANF and B-type natriuretic peptide (BNP). CREB-binding protein (CBP) and p300 co-activators were reported to have HAT activity and induce hypertrophic responses in cardiac muscle cells through treatment with phenylephrine (PE) [100]. After these reports, the contribution of core histone modifications to cardiac remodeling and also the target genes remained an extensive research field, considering that fetal gene expression is activated during this non-adaptive response.

Papait *et al*. [101] assessed histone modifications aiming to describe the epigenetic changes in adult cardiomyocytes in cardiac remodeling induced by transverse aortic constriction (TAC) by chromatin immunoprecipitation assay (ChIP), namely: three associated with activation of H3K9ac, H3K27ac, and H3K4me3 regulatory regions, a marker of the transcribed genes (H3K79me2), and three markers related to repression regions (H3K9me2, H3K9me3, and H3K27me3). Even though this full epigenetic screening demonstrated that genes involved in cardiac function were affected by TAC, a clearer understanding was necessary. Thus, Palomer *et al*. [102] carried out *in vivo* and *in vitro*models reinforcing the protective role of SIRT3 against cardiac fibrosis and inflammation. Treatment of AC16 linage (human cardiac cells) with TNFα increased FOS protein expression, a subunit of AP-1 (activation protein 1), which is associated with fibrosis during inflammation in the heart. Further assessment revealed that SIRT3 deacetylated H3K27 in the promoter region of FOS, leading to the repression of the gene expression [102].

Unlike the previous SIRT3 study, Gu *et al*. [103] reported that the polycomb protein PH19 reduced H3K36m3 and increased H3K27m3 of gene promoter of SIRT2 in cardiomyocytes and heart tissue treated with AngII, providing an exponential expression of ANF and BNP, and consequently cardiac hypertrophy. More recently, Funamoto *et al*. [104] evaluated acetylation in the isolated left ventricle primary cardiomyocyte cells stimulated by PE, and the results indicated hyperacetylation of H3K9 and H3K122 provided by p-300 in the promoter region of hypertrophic genes ANF, BNP and β-myosin heavy chain (β-MHC). Interestingly, H3K9 acetylation predominated in the hypertrophic stage, whereas H3K122 acetylation was enhanced in the HF stage [104].

Many publications regarding histone and DNA methylation have also highlighted the signature and outcomes in the development of cardiac hypertrophy and HF. For example, Kaneda *et al*. [105] published a study in which H3K4me3 and H3K9m3 were shown as epigenetic markers of HF in a heart hypertrophic model with cardiomyocytes of left ventricles from Dahl salt-sensitive rats. Modifications in H3 and H4 in mice hearts were also observed 8 weeks after inducing HF by TAC. ChIP indicated that the H3K4me2 histone marking in the Atp2a2 gene promoter, the SERCA2 gene, was significantly reduced in the TAC group, whereas there was an increase in the Myh7 gene promotor, the pβ-MHC corresponding gene. Additionally, ChIP analysis of the Atp2a2 gene promoter presented an elevated H3K36me2 level, resulting in recruiting DNMT1 and DNMT3b and the methyl CpG binding protein 2 (MeCp2). However, the opposite event was observed in the Myh7 gene promotor [106]. These reports indicated that specific epigenetic modifications are key contributors to the pathophysiology of cardiac hypertrophy and subsequent HF.

In relation to epigenetic modifications, CRS shows a restricted quantity of reports. As previously mentioned, uremia and inflammation participate in CRS pathophysiology. The increased homocysteine and S-adenosylhomocysteine levels in plasma are capable of inducing DNA hypomethylation and atherosclerosis. This evidence indicates that uremia provides cardiac epigenetic modification after kidney injury [107]. Inflammation was also associated with aberrant DNA methylation, however more robust data are lacking [108]. Gaikwad *et al*. [109] reported that acetylation at H3K9 and H3K23, dimethylation at H3K4 and H3K9, and phosphorylation at H3K10 were exacerbated in hearts during unilateral nephrectomy in mice with diabetic cardiomyopathy. As consequence, several genes involved in the cardiac remodeling were overexpressed in the heart of renal failure and diabetic mice, such as myosin heavy chains 3, 6, and 7, myosin light chain 3, metalloproteinase 1, laminin-β2, tubulin-α, plasminogen, etc. More recently, Huang *et al*. [110] executed CRS type 4 *in vivo* and *in vitro* models, when it was reported that hyperphosphatemia leads to hyperacetylation of H3K9 by histones acetyltransferases p300, CREB, and HAT1 in the promoter region of the transcription factor interferon regulatory factor 1 (IRF1). IRF1 inhibited the expression of PCG-1α, resulting in cardiac mitochondria dysfunction and metabolic changes [110]. Even though it is clear that kidney injury may yield histone modifications in the heart, specific reports in the CRS type 3 do not exist so far, being an open field to be examined.

All these findings over the last few years let to presume that both miRNAs and epigenetics modifications may be extensively studied in AKI patients in order to predict further heart complications and possibility interrupt CRS 3 development.

## 3. Kidney and Heart Conversation: Role of EVs 

EVs are defined as heterogeneous membrane vesicles secreted by several cell types having intercellular communication as their main function, either in physiological or pathological conditions, and transporting a large diversity of cargo, such as DNA, mRNA, miRNAs, protein, metabolites, and lipids. EVs are generally classified into two major groups according to biogenesis and size, namely exosomes and microvesicles [111]. Exosomes are derived from intraluminal vesicles (ILVs) in multivesicular bodies (MVB) which are formatted through internal budding of the endosomal membrane and released to extracellular space when MVB fuse upon the plasma membrane. Microvesicles originate from outward budding and fission of the plasma membrane, and are released into the extracellular space [112]. The size of these particles may vary from about 30 to 1000 nm or more; exosomes have a diameter range of 30–150 nm, whereas microvesicles have a range from 50–1000 nm. Once released, EVs reach the target cells, bind, or fuse to them, and may modulate the cellular processes [111].

EVs are frequently reported in pathological conditions, especially as biomarkers. A large diversity of AKI models has reported a massive type of urinary extracellular vesicles (uEV) biomarkers to predict the development of AKI before the traditional creatinine increased detection and oliguria [113] due to the easy and economic collection of urine, which is a non-invasive procedure [114, 115]. All nephron segments may release EVs containing specific markers, indicating the cell origin. Some of these markers are the kidney-specific marker, cluster of differentiation 24 (CD24), and the nephron segment markers aquaporin-1, aquaporin-2, Podocin, and Type 2 Na-K-2Cl, which are located in EVs from proximal tubular cells, collecting ducts, podocytes, and the thick ascending limb of Helen’s loop, respectively [114]. Another usual protein biomarker involved in the development of AKI is ATF3, reported for being increased in the early stage of AKI [116]. Furthermore, EVs play a crucial role in AKI associated with their prothrombotic, proinflammatory, and immunomodulatory properties [117], such as those demonstrated by Lv *et al*. [49] in an *in vivo* model of Lipopolysaccharide (LPS) and Adriamycin-induced AKI, when the increase of exosomal miRNA-19b-3p induced the macrophage phenotype M1 and led to tubular inflammation. This evidence indicates the potential role of EVs in AKI diagnosis and prognosis.

EVs may be key players in the pathophysiology of cardiac remodeling/hypertrophy and HF through the regulation of different pathways according to stimuli and the transported cargo, as demonstrated by some *in vitro* studies in which cardiomyocytes enhance the secretion of EVs after oxidative stress, hypoxia, inflammation, and treatment with heat shock protein 60 (HSP 60), leading other cardiomyocytes to cell death [118]. The increased release of EVs containing proinflammatory cytokines from cardiomyocytes in MI is associated with activation and infiltration of innate immune cells and more secretion of proinflammatory cytokines and chemokines resulting in cardiac remodeling and dysfunction [119]. Another emerging pathological role of EVs in HF is the regulation of redox status which target antioxidant agents, like nuclear erythroid factor 2 (Nrf2) and SOD, such as miRNA-1, miRNA-7, and mi-RNA-28a [119, 120]. Another interesting emerging role of EVs in cardiovascular diseases is their interplay with autophagy and also their ability to induce autophagy implicating adaptation and protection from HF [121].

Although EVs are strictly related to kidney and heart diseases in a such way that they may be isolated from plasma and serum as predictable circulating biomarkers, much research is needed to understand the participation of these particles in CRS [39]. Exosomes have been reported to be involved in the crosstalk between heart and kidney, especially in chronic heart and renal diseases. In a systematic review, Mas-Bargues *et al*. [122] grouped different evidence demonstrating that EVs from leukocytes, platelets, and endothelial cells participate in the etiopathogenesis of cardiovascular diseases in CKD, and the regulation of the biogenesis, release, and uptake may be an effective therapeutic approach to mitigate both kidney and heart injury. EVs released from senescent cells in chronic kidney disease associated with cardiovascular disorders are also emerging biomarkers of kidney and heart dysfunction in aging [122, 123] Verbree-Willemsen *et al*. [124] showed that EVs containing cystatin C and cluster of differentiation 14 (CD14) are associated with both kidney dysfunction and HF in dyspneic patients. A converse point about EVs in the kidney-heart communication was demonstrated in a MI study conducted by Gao *et al*. [125], in which exosomes containing miRNA-1956 likely released from ischemic myocardium and kidney after MI induction may lead to activating adipose-delivery mesenchymal stem cells, which are important cells for tissue regeneration. In summary, EVs are emerging tools to be explored for diagnostic, prognostic, and therapeutic aspects in the CRS context.

## 4. New Challenges in CRS 3 Diagnosis 

### 4.1 Potential Biomarkers and Technologies in the Diagnosis of AKI and HF

According to Kulvichit *et al*. [126], the continuum of AKI from initial kidney stress to an early injury, and then to dysfunction and long-term outcomes is the current conceptual model of the clinical course of AKI, wherein biomarkers at each point of the continuum may help define the mechanisms and predict the evolution of AKI.

Traditional biomarkers for assessing kidney function include serum creatinine, albuminuria, and cystatin C, as well as urine output and eGFR [127]. In any case, due to the limitations of serum creatinine and the complexity of AKI, a great effort has been made to develop biomarkers to improve the diagnosis and management of AKI. According to the AKI continuum, biomarkers can be broadly classified into two main categories: damage markers and dysfunction markers, such as human neutrophil gelatinase-associated lipocalin (NGAL), KIM-1, liver-type fatty acid–binding protein (L-FABP) and C-C motif chemokine ligand 14 (CCL14) as damage markers; proenkephalin amino acids 119 through 159 (penKid) as a function marker, and as stress tissue inhibitor of metalloproteinases-2 (TIMP2) and insulin-like growth factor–binding protein 7 (IGFBP7) [126].

Novel serum, plasma, and urinary AKI biomarkers have been discovered with the use of transcriptomic and proteomic technologies, and they have also garnered excitement; however, their adoption into routine clinical care has been slow due to the multi-factorial availability of testing platforms, cost, variability in assay techniques and results, as well as a lack of approval from national and international governance bodies [128].

Interestingly and similarly, the search for an ideal AKI biomarker was once viewed to be similar to the discovery of cardiac troponin for MI, but with a multifactorial cause [126]. HF biomarkers can be classified into several categories: biomarkers associated with myocardial and vascular stretching, such as BNP, NT-proBNP, troponins; biomarkers that reflect an alteration in the neurohormonal pathways and RAAS; biomarkers seen in inflammation and the oxidative process; and finally, biomarkers associated with myocardial and vascular fibrosis, such as suppression of tumorigenicity 2 (ST2) and galactin 3 [129].

As described by Biasucci *et al*. [130], novel biomarkers in HF may support the routinely used traditional ones by improving diagnosis and prognosis and thereby enhancing patient care. Thus, there is a growing interest in the multi-marker approaches because of their benefit over single biomarkers to increase diagnostic accuracy and to improve risk stratification in HF. miRNAs have greater stability and stronger targeting compared to traditional diagnosis and treatment, as presented by Luo *et al*. [131], which is a potential strategy for the diagnosis and treatment of HF.

### 4.2 Current Proposes for CRS 3 Diagnosis

It is known that cardiac dysfunction can determine injury to the kidney and kidney injury will reciprocally affect both the circulatory system and the heart, as the causal relationship between CKD, cardiovascular risk, and HF has been demonstrated by clinical and epidemiological studies [132]. In this sense, many biomarkers have been proposed such as cystatin C, IL-6, IL-18, KIM-1, NGAL, and Netrin-1 (NTN1) [133]. A metabolomic study reported a shift in energy production in both the heart and kidney in an animal model of CRS 3, leading to oxidative stress. Besides the potential therapeutic applicability, these finds suggest that oxidative stress systemic biomarkers may be plausible diagnostic tools [134].

In the same way, mineral and bone disorder biomarkers such as fibroblast growth factor 23 (FGF-23) and vitamin D have been suggested to participate in CRS development; while mean platelet volume (MPV), hepcidin, soluble urokinase-type plasminogen activator receptor (suPAR), placental growth factor (PIGF), urinary cofilin-1, urinary adrenodoxin (ADX), eosinophil cationic protein (ECP), fetuin B (FETUB), growth differentiation factor 15 (GDF15), guanine deaminase (GUAD) and neurogenic locus notch homolog protein 1 (NOTCH1, urinary proteins) might be useful in prognostic CRS features [127]. In addition the participation in pathophysiology, miRNAs are also emerging biomarkers in the context of CRS. Ahmed *et al*. [135] carried out an *in silico* research of miRNAs in the CRS, in which hsa-miR-21-5p was found in 8 pathways out of principal 10, and more 5 miRNAs were prominent, hsa-miR-122-5p, hsa-miR-222-3p, hsa-miR-146a-5p, and hsa-miR-29b-3p. The research suggests that the found 6 miRNAs may be promising diagnostic biomarkers. Recently, Fan *et al*. [136] reported that three miRNAs were significantly downregulated in the serum of acute MI patients with subsequent AKI. miR-24, miR-23a, and miR-145 were associated with the regulation of TGF-β and apoptosis pathways. Another study with cardiac surgery patients with AKI demonstrated that miR-21 in plasma, and specially in urine, may predict future renal complications, prolonged stay in hospital as well as high mortality [137].

Vibrational spectroscopy has become an important tool in biomedical analysis considering its specificity and sensibility using biological samples. Given the potential to provide essential information on the composition and structural conformation of specific molecular species, Fourier Transform (FT) Infrared and Raman spectroscopies have been explored in several diagnostic experiments [138, 139, 140]. FT-Raman spectroscopy has also been used to study CRS 3 induced by renal IR in an animal model by [140], in which bands related to tyrosine and tryptophan (which are the main raw materials of the protein-bounded uremic toxins) were found. Recent studies have also shown that infrared spectroscopy can be successfully adapted to experimental practice to analyze EVs [138].

**Fig. 2.**
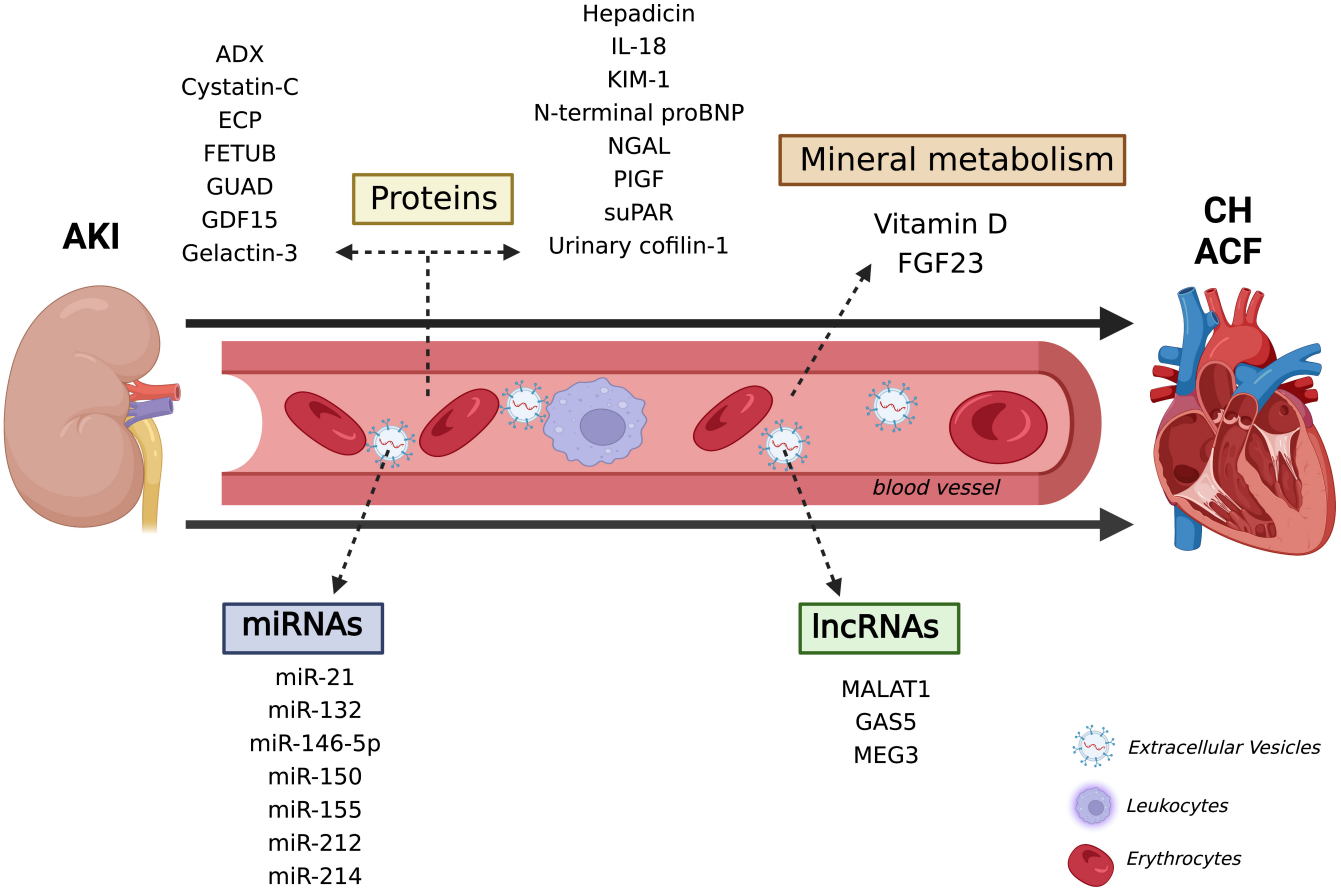
**New pathophysiological mechanism and promising molecules in the CRS 3**. Several types of molecules have been indicated to contribute to kidney and heart diseases, from nucleic acids, such as miRNAs, to proteins and vitamin D. There is increasing consent that these molecules are also associated with AKI-acute heat failure axis and confer promising biomarkers for diagnosing and monitoring CRS 3. ACF, Acute cardiac failure; ADX, urinary adrenodoxin; AKI, Acute kidney injury; CH, Cardiac hypertrophy; ECP, Eosinophil cationic protein; FETUB, Fetuin B; FGF23, Fibroblast growth factor 23; GAS5, Growth Arrest Specific 5; GUAD, Guanine deaminase; GDF15, Growth differentiation factor 15; IL-18, Interleukin 18; KIM-1, Kidney injury molecule 1; lncRNAs, long non-coding RNAs; MALAT1, Metastasis-associated-related lung adenocarcinoma transcript 1; MEG3, Maternally expressed 3; miRNAs, micro RNAs; NGAL, Neutrophil gelatinase-associated lipocalin; PIGF, Placental growth factor; suPAR, Soluble urokinase-type plasminogen activator receptor.

Taking into account all the new mechanisms and biomarkers pointed out in this systematic review regarding both kidney and heart disease isolated studies, as well as CRS research, it is proposed that these molecules may be directly related to the AKI-acute HF axis, and even seem to be promising tools to diagnose and monitor the CRS 3 pathophysiological course (Fig. 2). Besides the discovery of promising biomarkers by high-throughput technologies, such as transcriptomic and proteomic, in animals and cellular models, the application in the clinical is still elusive and needs a better evaluation.

## 5. Conclusions

The present review showed the timeline since the past to the future possibilities of understanding how the kidney and heart are closed connected and how the interferences can lead to different types of CRSs. Although much is known about the kidney-heart axis connectors, new experimental strategies have emerged with the aim of optimizing diagnosis and treatment in different heart and kidney disease scenarios in order to meet the challenges of precision medicine 4.0.

## Abbreviations

α-MHC, α-myosin heavy chain; α-SMA, α-smooth muscle actin; β-MHC, β-myosin heavy chain; ACE, angiotensin-converting enzyme; ACF, Acute cardiac failure; ADX, Urinary adrenodoxin; AKI, Acute kidney injury; ANF, Atrial natriuretic factor; Ang II, Angiotensin II; AP-1, Activation protein 1; ARC, Apoptosis repressor with caspase recruitment domain; ATF3, Activating transcription factor 3; ATR1, Angiotensin receptor 1a; BNP, B-type natriuretic peptide; CBP, CREB-binding protein; CCL14, C-C motif chemokine ligand 14; CH, Cardiac hypertrophy; ChIP, Chromatin immunoprecipitation assay; CD14, cluster of differentiation 14; CD24, cluster of differentiation 24; circRNAs, circular RNAs; CKD, Chronic kidney disease; CRS, Cardiorenal syndrome; DAMPs, Damage-associated molecular patterns; DNMT, DNA Methyltransferases; ECP, Eosinophil cationic protein; eGFR, Estimated glomerular filtration; ESRD, End-stage renal disease; EVs, Extracellular vesicles; FBXW7, F-box and WD-40 domain protein 7; FETUB, Fetuin B; FGF-23, Fibroblast growth factor 23; FT, Fourier Transform; G6PD, Glycose-6-phosphate dehydrogenase; GAS5, Growth Arrest Specific 5; GDF15, Growth differentiation factor 15; GUAD, Guanine deaminase; HDAC, Histone deacetylase; HF, Heart failure; HIF-1α, Hypoxia inducible factor 1α; HMGA1, High mobility group A1; HSP 60, Heat shock protein 60; IL-1β, Interleukin 1β; IL-6, Interleukin 6; IL-18, Interleukin 18; ILVs, Intraluminal vesicles; IFN- γ, Interferon γ; IGFBP7, Insulin-like growth factor–binding protein 7; IR, Ischemia and reperfusion; IS, Indoxyl sulfate; KIM-1, Kidney injury molecule-1; KLK1, Kallikrein1; L-FABP, Liver-type fatty acid–binding protein; LPS, Lipopolysaccharide; lncRNAs, long non-coding RNAs; MALAT1, Metastasis-associated-related lung adenocarcinoma transcript 1; MeCp2, Methyl CpG binding protein 2; MEF2, Myocyte enhancer factor-2; Mfn2, Mitofusin-2; MEG3, Maternally expressed 3; MI, Myocardial infarction; MPT, Mitochondrial permeability transition; MPV, Mean platelet volume; mRNAs, Messenger RNAs; MVB, Multivesicular bodies; ncRNAs, Non-coding RNAs; NF‑κB, Nuclear factor kappa beta; Nrf2, Nuclear erythroid factor 2; NGAL, Neutrophil gelatinase-associated lipocalin; NOTCH1, Neurogenic locus notch homolog protein 1; NT-proBNP, N-terminal proBNP; NTN1, Netrin-1; PAI-1, Plasminogen activation inhibitor 1; PE, Phenylephrine; penKid, Proenkephalin amino acids 119 through 159; PIGF, Placental growth factor; PCS, P-cresyl sulfate; RAAS, Renin-angiotensin-aldosterone system; RCS, Renocardiac syndrome; ROS, Reactive oxygen species; SIRT3, Sirtuin 3; SNS, Sympathetic nervous system; SOD2, Superoxide dismutase 2; SOCS, Suppressor of cytokine signaling‑1; ST2, Suppression of tumorigenicity 2; suPAR, Soluble urokinase-type plasminogen activator receptor; TAC, Transverse aortic constriction; TGFβ, Transforming growth factor beta; TIMP2, Tissue inhibitor of metalloproteinases-2; Thrap1, Thyroid hormone–associated protein 1; TLRs, Toll-like receptors; TSP1, Thrombospondin 1; TWEAK, Tumor necrosis factor-like weak inducer of apoptosis; uEV, Urinary extracellular vesicles; VCP, Valosin-containing protein.
